# Altered stability of dynamic brain functional architecture in primary open-angle glaucoma: a surface-based resting-state fMRI study

**DOI:** 10.1007/s11682-023-00800-7

**Published:** 2023-10-19

**Authors:** Bingbing Yang, Mingyue Su, Qian Wang, Xiaoxia Qu, Huaizhou Wang, Weiwei Chen, Yunxiao Sun, Ting Li, Yang Wang, Ningli Wang, Junfang Xian

**Affiliations:** 1 Department of Radiology, Beijing Tongren Hospital, Capital Medical University, No.1 of Dongjiaominxiang Street, Dongcheng District, Beijing, 100730 China; 2 Beijing Tongren Eye Center, Beijing Tongren Hospital, Capital Medical University, No.1 of Dongjiaominxiang Street, Dongcheng District, Beijing, 100730 China; 3 Department of Radiology, Medical College of Wisconsin, Milwaukee, WI USA

**Keywords:** Functional stability, Dynamic functional connectivity, Resting-state functional magnetic resonance imaging, Primary open-angle glaucoma

## Abstract

Delineating the neuropathological characteristics of primary open-angle glaucoma (POAG) is critical for understanding its pathophysiology. While temporal stability represents a crucial aspect of the brain's functional architecture, the specific patterns underlying its contribution to POAG remain unclear. This study aims to analyze the brain functional abnormalities in POAG using functional stability, a dynamic functional connectivity (DFC) approach based on resting-state functional magnetic resonance imaging (rs-fMRI). Seventy patients with POAG and forty-five healthy controls underwent rs-fMRI and ophthalmological examinations. The stability of DFC was calculated as the concordance of DFC over time using a sliding-window approach, and the differences in stability between the two groups were compared. Subsequently, Spearman’s correlation analyses were conducted to examine the relationship between functional stability and clinical indicators. Compared with healthy controls, patients with POAG exhibited significantly decreased functional stability in the visual network, including the early visual center, ventral and dorsal stream visual cortex in both hemispheres. Conversely, stability values increased in the bilateral inferior parietal gyrus and right inferior frontal cortex. In POAG patients, the dynamic stability of the left early visual cortex and ventral stream visual cortex correlated with the mean deviation of visual field defects (*r* = 0.251, *p* = 0.037). The evidence from this study suggests that functional stability may provide a new understanding of brain alterations in the progression of POAG.

**Supplementary Information:**

The online version contains supplementary material available at 10.1007/s11682-023-00800-7.

## Introduction

Glaucoma stands as the leading cause of irreversible blindness worldwide. Nevertheless, primary open-angle glaucoma (POAG), being the most prevalent type of glaucoma, encounters challenges in its pathogenesis, diagnosis, and management (Jonas et al., 2017; Stein et al., 2021; Tham et al., 2014). The mounting body of neuroimaging studies has consistently indicated that POAG is a neurodegenerative disease characterized by a hidden onset and a high rate of missed diagnosis (Jonas et al., 2017; Mutlu et al., 2018; Sendi et al., 2021), with symptoms typically remaining unnoticed, slowly progressing, and ultimately leading to irreversible blindness upon detection (Casson et al., 2012; Stein et al., 2021). Additionally, patients are often diagnosed at an intermediate or advanced stage, lacking effective intervention and treatment options (Jonas et al., 2017). Therefore, achieving an early diagnosis of glaucoma is pivotal for prognosis, but it remains challenging due to the elusive pathogenesis of POAG (Chan et al., 2021). Consequently, it is imperative to identify neuroimaging biomarkers capable of monitoring disease progression and shedding light on the underlying pathogenesis of POAG.

Resting-state functional magnetic resonance imaging (rs-fMRI) has received considerable scholarly attention in the field of functional neuroimaging. Recent literature has increasingly reported extensive abnormal changes in the visual cortex and other related brain areas in patients with POAG based on rs-fMRI. Studies investigating the amplitude of low-frequency fluctuation (ALFF) and regional homogeneity (ReHo) have demonstrated abnormal local spontaneous brain activity in visual, sensory, motor, and cognitive brain regions in patients with POAG (Li et al., 2014, 2021; Song et al., 2014). Distinct from local features of spontaneous brain activity that only characterize local temporal coherence, functional connectivity (FC) can capture the temporal coherence of spontaneous brain activity between spatial regions (Biswal et al., 1995; Fox & Raichle, 2007). Thus far, the findings of FC alteration in glaucoma are still controversial. Some studies have indicated that no significant functional connectivity disruptions were observed either within or between functional networks in glaucoma (Demaria et al., 2022; Wang et al., 2016). However, certain other studies have found functional changes in vision-related brain regions in glaucoma, by investigating FC within specific networks rather than across different networks. These investigations have revealed decreased FC in visual areas (Wang et al., 2021b; Dai et al., 2013; Wang et al., 2017), as well as alterations within brain regions linked to working memory and cognitive functions in patients with POAG (Wang et al., 2021a, b; Dai et al., 2013; Wang et al., 2020). Furthermore, a study on voxel-mirrored homotopic connectivity in POAG identified decreased interhemispheric homotopic FC between the bilateral visual cortex in POAG patients (Wang et al., 2018). Additionally, alterations in brain structure, blood perfusion, and metabolism within and outside visual pathways and other brain regions have been increasingly recognized in POAG patients (Frezzotti et al., 2016; Wang et al., 2021b; Mendoza et al., 2022; Zikou et al., 2012; Guo et al., 2018). However, these neuroimaging indices primarily reflect static characteristics of spontaneous brain activity, contrasting with the concept of time-dependent dynamic characteristics of spontaneous neural brain activity during rest (Vidaurre et al., 2017; Hutchison et al., 2013; Allen et al., 2014; Chang & Glover, 2010). Hence, investigating the dynamic properties of brain alterations represents a potential avenue to elucidate the intricate brain function at different timescales.

While brain function is inherently dynamic, regulating the functional stability of different brain regions in a continuous or flexible state is crucial for normal brain function (Li et al., 2020; Liégeois et al., 2017). Stability plays a vital role in consciousness, relating to the efficient specialization or rapid switching of representation of information through distributed neural activity and connectivity patterns over time. The physiological mechanisms underlying functional stability and the hierarchical organization of stability across different brain regions remain contentious. Some researchers proposed that areas of higher-order association exhibit higher temporal variability compared to unimodal regions (Yin et al., 2016; Zhang et al., 2016). This could be due to higher-order association regions coordinating different information types and maintaining dynamic connections with various systems (Mesulam, 1998). However, contrasting viewpoints have also emerged, with some researchers suggesting that the functional organization of higher-order association regions may exhibit greater stability compared to that of unimodal regions (Kong et al., 2019; Laumann et al., 2015; Mueller et al., 2015). Higher stability values indicate more concordant and stable dynamic functional architecture configurations over time, whereas lower stability values imply a reduced capacity to coordinate information over time, suggesting that brain stability can rapidly transfer from one state to another (Li et al., 2020). To date, functional stability analysis has been employed in studies of neurological and psychiatric disorders, such as amyotrophic lateral sclerosis (Wei et al., 2021), major depressive disorder (Zhu et al., 2020), and cirrhotic patients with minimal hepatic encephalopathy (Cai et al., 2022), shedding light on the underlying mechanisms of various neuropsychiatric disorders and potentially serving as a biomarker for disease progression monitoring. However, the stability of brain functional organization in POAG remains unexplored. To investigate the functional stability alteration within the cerebral cortex, we utilized a surface-based analysis method that is well-aligned with our research focus. A recent validation and publication have demonstrated that this method's efficacy is nearly three times better than the traditional volume-based approach (Coalson et al., 2018; Espinoza et al., 2019).

Thus, the objective of this study was to investigate alterations in functional stability in the brain of patients with POAG based on rs-fMRI data. We hypothesized that POAG patients would exhibit significant alterations in dynamic functional stability, and we further hypothesized that these alterations would correlate with disease severity. To test our hypotheses, we employed dynamic stability analysis using the surface-based method, providing a novel perspective to expand the current understanding of the neuropathological underpinnings of POAG.

## Methods

### Participants

Informed consent was obtained from all participants in accordance with the Declaration of Helsinki, and the study was approved by the Medical Ethics Committee of Beijing Tongren Hospital. A total of 71 POAG patients were recruited from both outpatient and inpatient settings at our hospital. Forty-six healthy controls were recruited locally through recruitment advertising. All subjects were right-handed. The inclusion criteria for POAG patients were as follows: (1) age between 20 and 75 years; (2) a clinical examination confirming POAG (Prum et al., 2016); (3) the presence of glaucomatous damage to the optic nerve and glaucomatous VF defect; and (4) mean deviation ≥  − 12 dB. The examination results were assessed in a blinded manner by three glaucoma specialists. Participant enrollment in the study required unanimous agreement from all three specialists regarding the diagnosis. The average visual field (VF) mean deviation (MD) for both eyes was utilized as a comprehensive index to assess the overall status of visual field function. Furthermore, it was employed for correlation analysis with changes in brain imaging. Healthy controls without clinical evidence or history of glaucoma were matched to the POAG patients in terms of age and sex. All control participants underwent visual acuity assessments, intraocular pressure measurements, slit-lamp examinations, and fundus examinations, all of which were conducted by specialized glaucoma ophthalmologists, to ensure that they did not have a diagnosis or suspicion of glaucoma. The exclusion criteria for both groups included: (1) clinical evidence or history of other eye diseases; (2) visual fields with false negative response rate > 33%, false positive response rate more than 15% and fixation loss rate > 20%; (3) significant psychiatric, neurological, or systemic comorbidities; (4) abnormal signals in visual pathways or brain images on magnetic resonance imaging (MRI); and (5) contraindications to MRI scans.

### MRI data acquisition

MRI data were acquired using a 3.0-T MR scanner (Discovery MR750; General Electric, Milwaukee, WI) with an 8-channel head coil. T1-weighted images and rs-fMRI data were obtained from all participants. Structural 3D T1-weighted images were acquired with the following parameters: repetition time (TR) = 8.16 ms, echo time (TE) = 3.18 ms, inversion time (TI) = 450 ms, flip angle = 12°, matrix = 256 × 256, slice thickness = 1.0 mm, gap = 0 mm, slices = 188, and voxel size = 1 × 1 × 1 mm^3^. The rs-fMRI data were acquired using a gradient-echo single-shot echo planar imaging sequence with the following parameters: TE = 30 ms, TR = 2000 ms, slice thickness = 3 mm, gap = 1 mm, flip angle = 90°, field of view = 220 × 220 mm^2^, matrix = 64 × 64, axial slices = 36 and time points = 180. During the scan, all participants were asked to lie still with their eyes closed and allow their minds to wander. To minimize head movement and reduce scanner noise, foam fillers and earplugs were utilized, respectively.

### Rs-fMRI data preprocessing

All imaging data were preprocessed using DPABISurf 6.0, a surface-based rs-fMRI data analysis toolbox (Yan et al., 2021), based on DPABI (Yan et al., 2016), fMRIPrep (Esteban et al., 2019), ANTs (Avants et al., 2008), FreeSurfer (Dale et al., 1999), SPM (Ashburner, 2012), MATLAB 2018a (The MathWorks Inc., Natick, MA, US), and Docker (https://docker.com).

The preprocessing pipeline in this study involved the following steps: (1) removal of the initial 10 time points to allow for signal equilibration; (2) conversion of the data into Brain Imaging Data Structure (BIDS) format (Gorgolewski et al., 2016), followed by calling the fMRIPrep 1.5.0 Docker; (3) structural image preprocessing, including intensity correction, skull stripping, spatial normalization, brain tissue segmentation, and surface reconstruction; (4) functional data preprocessing, which included brain mask generation, head motion estimation, slice timing correction, multiple linear regression (Yan et al., 2013; Zuo et al., 2013), and alignment of spatial correspondences to individual structural images using a boundary-based registration algorithm (Greve & Fischl, 2009). The blood oxygen level-dependent (BOLD) time series were resampled to surfaces on the fsaverage5 space; (5) nuisance regression, wherein the Friston 24-parameter model (Friston et al., 1996) was used to regress out head motion confounds. Additionally, mean framework displacement was used to address the residual effects of motion in group analyses (Jenkinson et al., 2002). Other sources of spurious variance (signals of white matter and cerebral spinal fluid) were also removed from the data through linear regression to reduce respiratory and cardiac effects. Furthermore, linear trends were included as a regressor to account for drifts in the BOLD signal; (6) application of a bandpass temporal filter (0.01–0.1 Hz) to the normalized functional images. Considering the sensitivity of rs-fMRI data to head motion (Van Dijk et al., 2012), two subjects (one patient with POAG and one healthy control) with maximum head motion exceeding 2.5 mm in displacement or 2.5° in rotation were excluded from subsequent analyses. Ultimately, 70 patients with POAG and 45 healthy controls were included in the study based on quality control criteria.

### Functional stability calculation

**Fig. 1:**
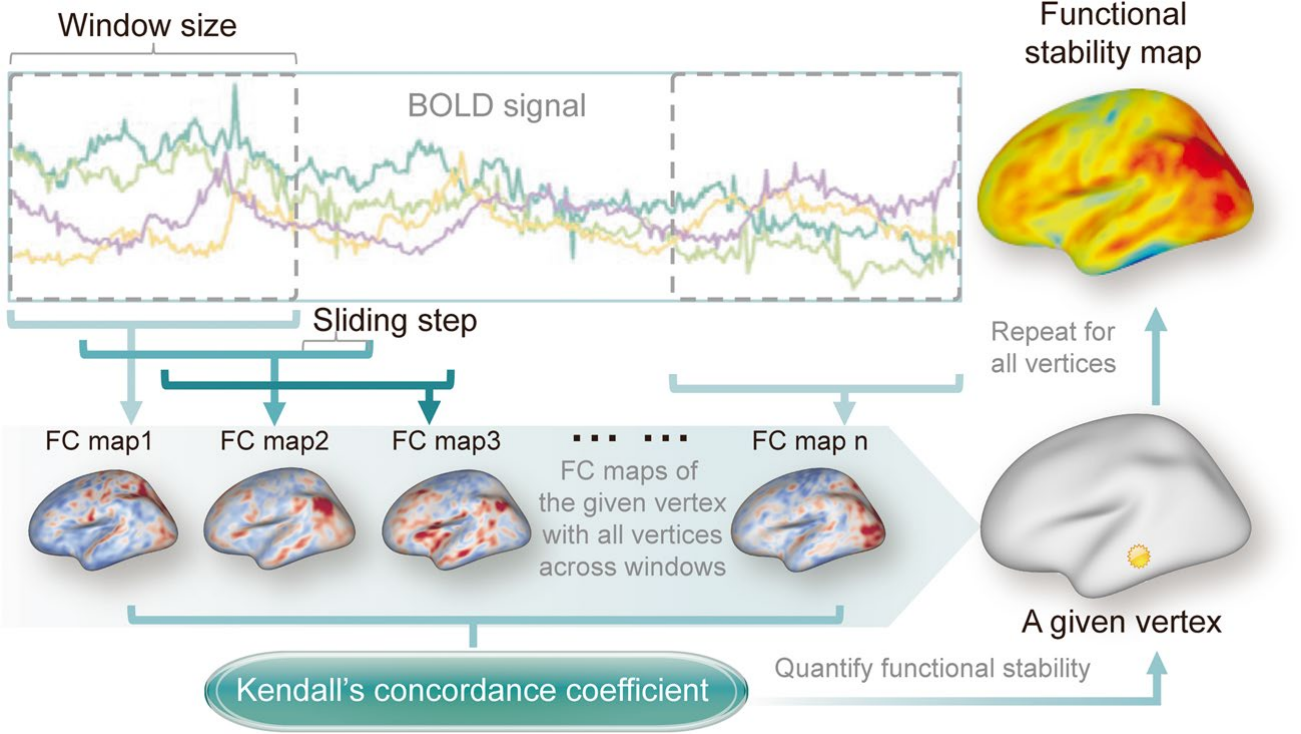
Schematic summary of the calculation procedure for surface-based functional stability. Initially, a range of dynamic FC maps was computed for a given vertex using a sliding-window approach. For instance, FC map 1 was obtained by calculating Pearson's correlation coefficients between the BOLD time series of the given vertex and those of all other vertices within the cortex mask during the first window. Subsequently, the functional stability of that vertex was quantified using Kendall's concordance coefficient based on these dynamic FC maps (FC map 1-n). Finally, by repeating the above-described procedure for all vertices within the cortex mask, a dynamic functional stability map was generated. BOLD, blood oxygen level-dependent; FC, functional connectivity

The within-state stability of dynamic functional architecture was defined as the concordance of dynamic functional connectivity (DFC) over time (Li et al., 2020) and was processed using the Stability Analysis module in the DPABISurf 6.0 toolbox (Yan et al., 2021). The calculation procedure for functional stability is summarized in Fig. 1. Specifically, for a given vertex, a range of DFC maps for that vertex was computed using a sliding-window approach with a window size of 64 s and a step size of 4 s (Li et al., 2020). FC maps were obtained by calculating Pearson's correlation coefficients between the BOLD time series of the given vertex and those of all other vertices within the cortex mask across the sliding windows. Subsequently, the functional stability of that vertex was quantified using the Kendall's concordance coefficient of these DFC maps. Finally, a dynamic functional stability map for a subject was obtained by repeating the above-described procedure for all vertices within the cortex mask. The vertex-by-atlas approach was applied to calculate the stability metric based on the Multi-modal Parcellation of Human Cerebral Cortex (i.e., HCP-MMP1.0 atlas) (Glasser et al., 2016). Once derived, the stability values of both hemispheres were further standardized to Z-scores by subtracting the mean and dividing by the standard deviation of the overall values within the cortex mask. Subsequently, Gaussian kernels of 6-mm full-width at half maximum were used for spatial smoothing on the fsaverage5 space. For a given vertex, a higher stability value indicates a less dynamic functional architecture configuration over time.

### Statistical analysis

Demographic and clinical data of all participants were analyzed using the Statistical Package for the Social Sciences version 25.0 (SPSS, IBM Inc., United States). The normality of demographic and clinical data was assessed using the Shapiro–Wilk test. For non-normally distributed data, such as ages and MD of VF defects, the median (range) was reported. The comparison of ages between groups was performed using the non-parametric Mann–Whitney U test, while the comparison of genders was conducted using the Chi-square test. The inter-eye MD VF difference in POAG patients was compared using the Wilcoxon Signed-rank test. The significance threshold was set at *p* < 0.05.

To compare the differences in stability between the two groups, a two-sample t-test was utilized. The Benjamini–Hochberg false discovery rate (BH-FDR) method was applied for multiple comparisons with the correction of *q* < 0.05 and a cluster size > 20mm^2^. The preserved clusters were labeled as regions based on the Multi-modal Parcellation of Human Cerebral Cortex (Glasser et al., 2016) and the Yeo atlas of cortex, which classifies regions according to resting-state networks derived from rs-fMRI (Yeo et al., 2011). As the brain surface was reconstructed and automatically divided into two hemispheres during preprocessing, statistical inference was performed separately for both hemispheres to address the multiple comparison issue. To validate the results, the cluster-wise threshold was adjusted to *q* < 0.025 to control the false positive rate through Bonferroni correction (0.05/2). Subsequently, Spearman’s correlation analyses were conducted between the functional stability values of the seven clusters and the MD of VF defects (BH-FDR corrected *q* < 0.05).

### Reproducibility analyses

As the parameter settings in the window-sliding method and the spatial smoothing process are still controversial, we conducted a reproducibility analysis. To examine the effects of different sliding-window parameters on the functional stability analyses, we performed between-group comparisons using various window sizes (50 s, 64 s, and 100 s) and sliding steps (2 s and 4 s) for the functional stability analyses. Additionally, to evaluate the impact of different smoothing procedures on our results, we employed various sizes of Gaussian kernels (6 mm, 8 mm, and 10 mm) for spatial smoothing.

## Results

### Demographics and clinical features

The clinical characteristics of the 70 patients with POAG and 45 healthy controls were listed in Table 1. There were no significant differences in age (*Z* = -1.425, *p* = 0.154) or sex *(χ*^2^ = 0.674, *p* = 0.933) between the POAG and healthy control (HC) groups. In addition, we found no significant difference in VF MD between the left and right eyes in POAG patients (*Z* = -0.876, *P* = 0.381).

**Table 1:** Demographic and clinical characteristics of POAG and HC groups

Variables	POAG	HC	*p* value
Sample size	70	45	–
Gender (male/female)	41/29	26/19	0.674^a^
Age (years)	40 (21 ~ 72)	37 (23 ~ 62)	0.154^b^
Handedness (R/L)	70/0	45/0	–
MD (dB)	−7.56 (−32.23 ~ 1.71)	–	–

Unless stated otherwise, data are presented as median (range). *POAG* Primary open-angle glaucoma; *HC* Healthy control; *MD* Mean deviation (an index of visual deficits derived from Humphrey visual field)

^a^ The *p*-value was assessed by the Chi-squared test

^b^ The *p*-value was assessed by the Mann-Whitney U test

### Case–control differences of the dynamic functional stability

**Fig. 2:**
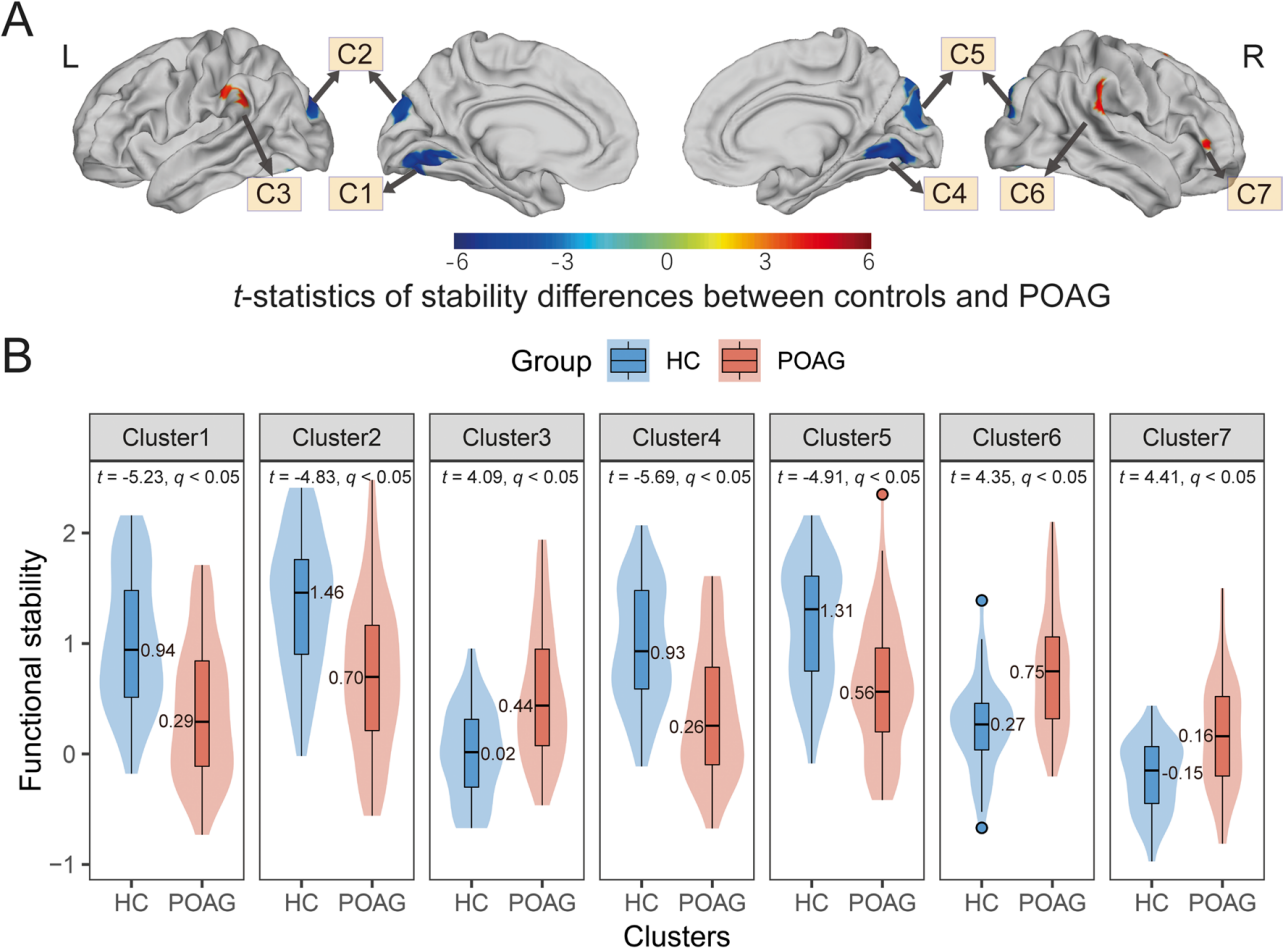
Dynamic functional stability differences between POAG patients and controls. **A** Case–control comparison (*t*-map) of dynamic functional stability, with warm colors representing POAG > HC and cold colors representing POAG < HC. Seven cortical clusters (C1-C7) showed statistically significant differences. **B** Violin and box plots depict the distribution and between-group differences of the mean value of dynamic functional stability in seven clusters. (Vertex-level *p* < 0.001; cluster-level *q* < 0.05, corrected by BH-FDR). L, left; R, right; POAG, primary open-angle glaucoma; HC, healthy control; BH-FDR, Benjamini–Hochberg false discovery rate

**Fig. 3:**
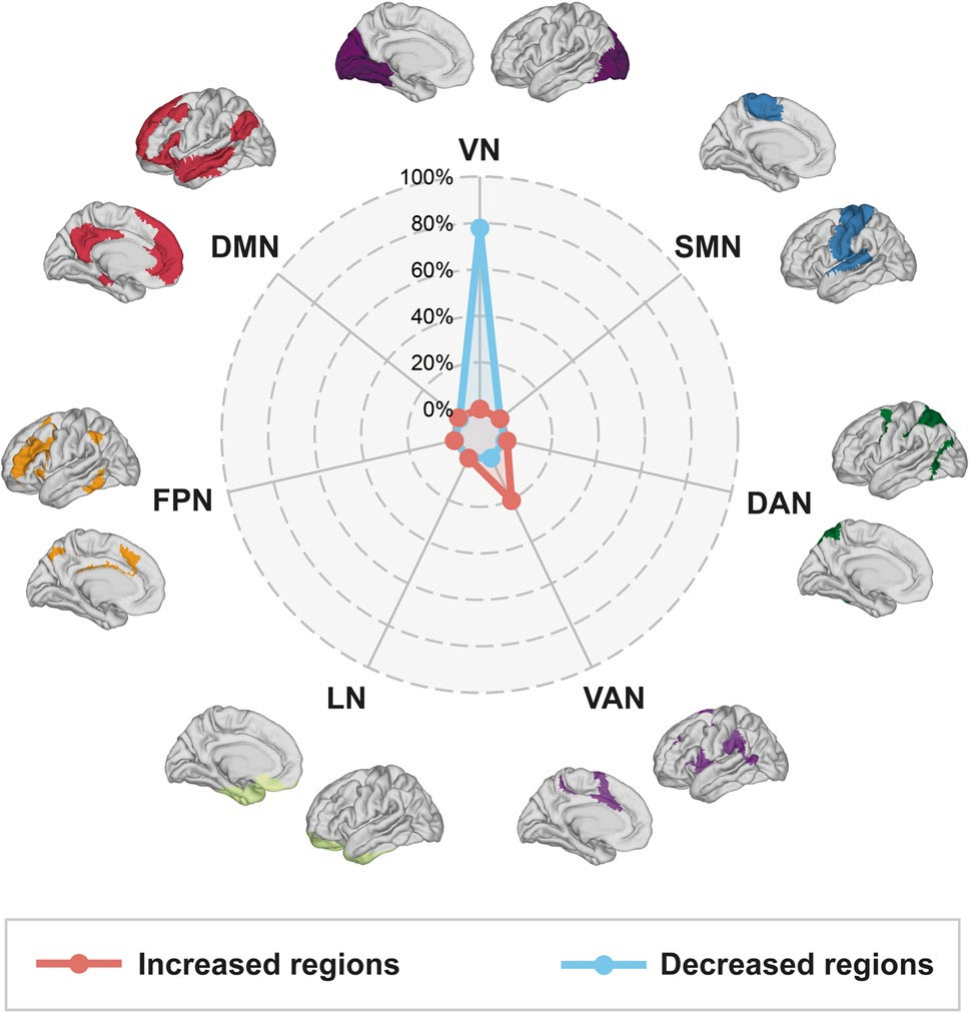
Distribution of statistically significant case–control differences according to functional networks (Yeo et al., 2011). Regions with increased/decreased functional stability in patients with primary open-angle glaucoma compared to healthy controls are shown in red/blue, respectively. VN, visual network; SMN, sensorimotor network; DAN, dorsal attention network; VAN, ventral attention network; LN, limbic network; FPN, frontoparietal network; DMN, default mode network

The differences of dynamic functional stability between the POAG patients and healthy controls are presented in Fig. 2 and Table 2 (uncorrected voxel-level *p* < 0.001, corrected cluster-level *q* < 0.05, BH-FDR corrected). Compared to healthy controls, POAG patients showed decreased functional stability in the visual network, including early visual centers (HCP-MMP-V2, V3, V4) (Glasser et al., 2016), ventral stream visual cortex (HCP-MMP-V8, PIT, FFC, VMV), and dorsal stream visual cortex (HCP-MMP-V3A, V3B, V6, V6A, V7, IPS) in both hemispheres, along with increased stability in the bilateral inferior parietal gyrus (HCP-MMP-PF, PFt, PFop, PFm) and the right inferior frontal cortex (HCP-MMP-IFSa). To validate the results, the cluster-wise threshold was adjusted to *q* < 0.025 for each hemisphere of the brain to address the issue of multiple comparisons arising from separate preprocessing of the left and right hemispheres. Our main findings remained consistent when applying a more stringent multiple comparison correction. (Fig. S1). To contextualize the regional case–control differences in stability, we referred them to the Yeo atlas of cortex classified according to resting-state networks (Yeo et al., 2011). Accordingly, the distribution of statistically significant case–control differences was described based on the Yeo network. In comparison to healthy controls, regions with decreased or increased stability in patients with POAG are mapped to visual network (VN) and ventral attention network (VAN), respectively (Fig. 3).

**Table 2:** Regions showing significant differences in stability between patients with POAG and healthy controls

Cluster	Location	L/R	Cluster size(mm^2^/Vertices)	Peak coordinates			Peak intensity(*t*)	Networks
				X	Y	Z		
1	Early visual cortex	L	663.633 / 120	15.1559	−66.8135	−35.487	−5.23082	VN
	Ventral stream visual cortex							
2	Early visual cortex	L	261.537 / 63	20.037	−94.7651	11.6763	−4.83415	VN
	Dorsal stream visual cortex							
3	Inferior parietal cortex	L	151.905 / 54	−38.6871	−17.9259	23.3727	4.09121	VAN, DAN
	Temporo-parieto-occipital junction							
	Posterior opercular cortex							
4	Early visual cortex	R	683.317 / 124	−12.1507	−71.6922	−37.7727	−5.69411	VN
	Ventral stream visual cortex							
	Primary visual cortex							
	Posterior cingulate cortex							
5	Dorsal stream visual cortex	R	407.476 / 104	−14.2656	−93.3304	13.2178	−4.90695	VN
	Early visual cortex							
	Posterior cingulate cortex							
6	Inferior parietal cortex	R	123.324 / 55	39.273	−28.3928	7.63864	4.35442	VAN
	Temporo-parieto-occipital junction							
7	Inferior frontal cortex	R	49.9233 / 16	21.5118	74.2133	−17.3296	4.40532	VAN, DMN, FPN

The statistical threshold was set at uncorrected voxel-level *p* < 0.001, corrected cluster-level *q* < 0.05 (BH-FDR corrected). *VN* Visual network; *DAN* Dorsal attention network; *VAN* Ventral attention network; *FPN* Frontoparietal network; *DMN* Default mode network; *BH-FDR* Benjamini–Hochberg false discovery rate

### Significant correlations between stability values of POAG and clinical features

**Fig. 4:**
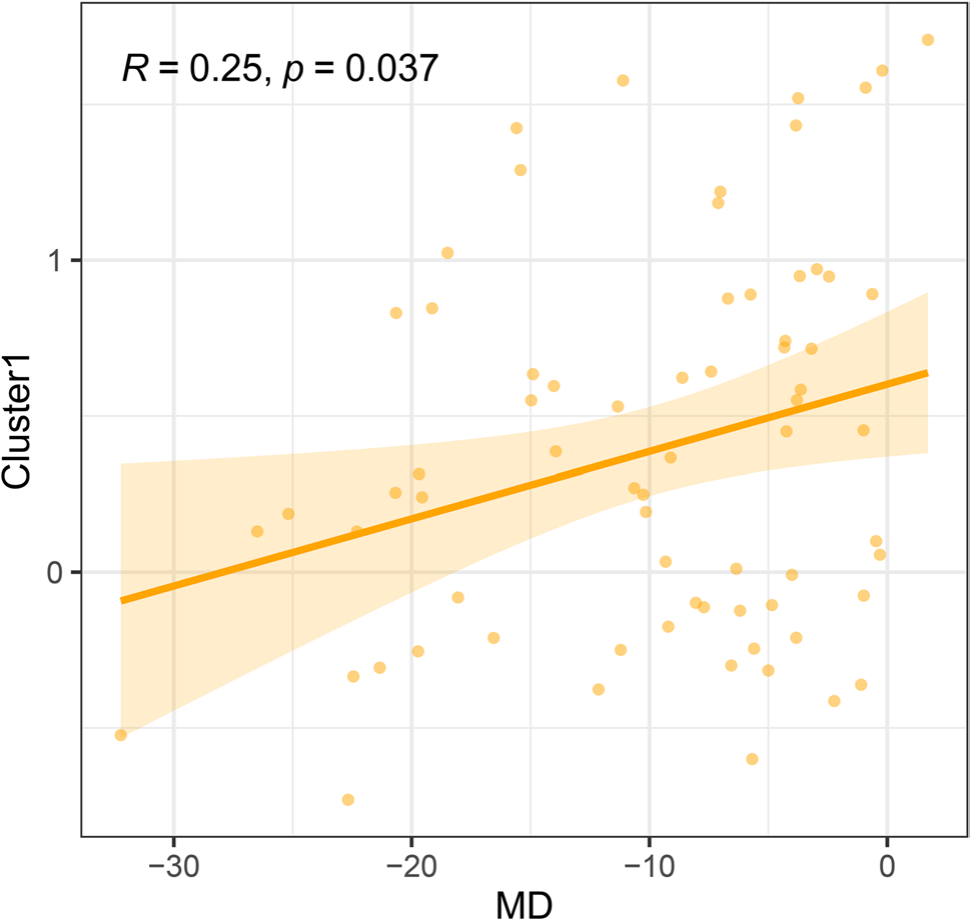
Significantly positive correlation between the MD of the VF defects and stability value of cluster 1 in patients with primary open-angle glaucoma (uncorrected *p* < 0.05). MD, mean deviation; VF, visual field

The results of Spearman’s correlation analyses between functional stability values of the seven clusters and MD of VF defects in patients are shown in Fig. 4 and Fig. S2. The cluster containing the left early visual cortex and ventral stream visual cortex (cluster 1) exhibited a slight positive correlation with the MD of VF defects in patients with POAG (*r* = 0.251, uncorrected *p* = 0.037) (Fig. 4). After BH-FDR correction (*q* < 0.05), no cluster showed a significant correlation with MD of VF defects (Fig. S2).

### Reproducibility analyses

Our main findings were reproducible when using functional stability derived from other combinations of window size and sliding step, indicating that variations in sliding windows had no significant impact on the results of our functional stability analyses (Fig. S3). Moreover, the analyses conducted with different Gaussian kernels in spatial smoothing demonstrated highly similar patterns of between-group differences in functional stability, validating the robustness of the primary results (Fig. S4).

## Discussion

In this study, we utilized rs-fMRI to analyze dynamic connectivity stability in POAG patients, revealing abnormal functional stability in their brains. Compared to the healthy subjects, patients with POAG exhibited decreased functional stability in the bilateral early visual center, ventral and dorsal stream visual cortex, whereas the stability of the bilateral inferior parietal gyrus and the right inferior frontal cortex increased. Moreover, we observed a significant correlation between the stability value in the left early visual cortex of patients with POAG and the MD of VF defects. Furthermore, we demonstrated that the results were consistent across different window-sliding and spatial smoothing parameters.

Stability is a fundamental attribute of consciousness, signifying the consistent and enduring representation of information achieved through distributed neural activity and connectivity patterns across various brain regions. The functional stability of brain voxels is defined as the persistency of their voxel-level dynamic functional connectivity (DFC) over the course of the scanning procedure (Li et al., 2020). Increased functional stability may suggest a constrained capacity of these regions to swiftly transition between distinct brain states or a compensatory mechanism for maintaining inter-regional information coordination. In contrast, reduced stability indicates an inability to sustain specialized information processing or an increased tendency for frequent and rapid shifts between separate brain states. We observed that the functional stability of VN regions (clusters 1, 2, 4, and 5) is greater than that of the VAN regions (clusters 3, 6, and 7) in healthy control participants, potentially indicating that higher-order association regions exhibit relatively lower stability in comparison to unimodal regions. This observation potentially suggests that higher-order association regions maintain dynamic connections with diverse systems to facilitate the coordination of distinct types of information. In contrast, unimodal regions tend to process information in a consistent manner. Abnormalities in functional stability have been investigated within neurological and psychiatric disorders, potentially offering novel insights into the fundamental mechanisms underlying these neuropsychiatric conditions.

Our study reveals a decline in the functional stability of the visual cortex in POAG, possibly leading to an inability to maintain a specialized and efficient visual information processing. This finding aligns with recent stimulus-driven fMRI investigations that have revealed reduced cortical activity in visual regions, exhibiting a correlation with disease severity among individuals with glaucoma (Carvalho et al., 2022). In addition, previous studies based on diffusion tensor imaging (DTI) findings have shown increased mean diffusivity and decreased fractional anisotropy in the anterior visual pathway and visual cortex in patients with POAG (Chen et al., 2013; Garaci et al., 2009; Zhang et al., 2012), indicating impairment of white matter fiber bundles in visual-associated brain regions. These findings suggest potential contributions to the observed alterations in dynamic functional stability. Duncan et al. (2012) demonstrated diminished cerebral blood flow (CBF) within the visual cortex in POAG, while Wang and colleagues (Wang et al., 2021b) further substantiated this by confirming a reduction in the coupling between CBF and functional connectivity strength in the visual cortical region, mirroring our findings of decreased stability. Furthermore, a clinicopathological study revealed cerebral degeneration of the intracranial optic nerve, lateral geniculate body (LGN), and visual cortex in human glaucoma patients (Gupta et al., 2006). Animal experiments have also demonstrated the presence of LGN and visual cortex lesions in experimental primate glaucoma models (Yücel et al., 2003). These studies may provide support for the degeneration of the visual cortex in glaucoma. However, the reduced cortical functional stability also suggests the possibility of frequent functional reorganization within visual region. Despite the reduced BOLD modulation, the population receptive field (pRF) estimates in early visual areas remained unaffected in all glaucoma participants, indicating the preservation of coarse retinotopic structure (Carvalho et al., 2022; Yildirim et al., 2018). This finding suggests that the adult brain maintains a certain level of local neuroplasticity within the visual cortex. Similarly, the observed reduction in stability within the visual cortex in this study could potentially be attributed to facilitating rapid adaptation to incoming external visual information.

Another important finding was the increased functional stability observed in the bilateral inferior parietal gyrus and right inferior frontal cortex in POAG patients. Visual information is transmitted through ventral and dorsal pathways. The former pathway extends to the temporal lobe and hippocampus and is responsible for processing color and shape information, while the latter involves the parietal, occipital, and frontal lobes, responding to spatial information and direction of motion (Maunsell et al., 1990; Stiers et al., 2006). Earlier investigations involving glaucoma models (Ito et al., 2009) and post-mortem analyses (Gupta et al., 2006) have suggested that elevated intraoptic pressure might potentially lead to impairments in these two pathways. These distinct pathways assume specialized roles in processing different aspects of visual information and are interconnected with the ventral attention network (VAN) and the dorsal attention network (DAN), which are functional networks involved in attention and visual processing (Solís-Vivanco et al., 2021). Imaging studies have previously reported decreased CBF in the bilateral inferior parietal gyrus (Wang et al., 2021b) and ReHo in the left inferior parietal gyrus (Song et al., 2014), indicating potential impairment in perfusion and function within these brain regions. Additionally, earlier investigations have documented a decline in gray matter volume in the bilateral inferior frontal gyrus (Chen et al., 2013), as well as microstructural damage in the left (Zikou et al., 2012) and right inferior fronto-occipital fasciculus (Giorgio et al., 2018). These alterations in brain regions may be associated with the abnormalities in reading (Swenor et al., 2017), facial recognition (Glen et al., 2012), and attention segmentation (Swenor et al., 2017) that previous studies have identified in patients with POAG. However, contrasting findings have emerged, indicating enlarged volumes of the bilateral inferior parietal gyrus, increased ALFF and blood flow in the left inferior parietal gyrus, and heightened FC in the bilateral VAN among glaucoma patients. These observations may align with the increased VAN stability identified in our study, reflecting a compensatory response. Interestingly, predictive masking could indicate localized neural plasticity in glaucoma patients, manifested as enduring cortical reorganization (Carvalho et al., 2021, 2022). These local changes may be interpreted as attempts by the visual system to compensate for losses caused by neural damage (Carvalho et al., 2022; McDonald et al., 2022). The process of predictive masking may involve ongoing predictions regarding target positions and conditions, potentially linked to the increased functional stability of the VAN, aiding in the sustained coordination of information transmission between the visual network and other brain regions. Our study reveals a lateralized increase in functional stability of the right inferior frontal gyrus in POAG patients. While our study found no significant difference in VF MD between the left and right eyes in POAG patients, subtle variations in individualized visual function across hemispheres cannot be entirely ruled out. Furthermore, lateralized effects are evident in various neurological conditions, indicating potential hemispheric specialization and susceptibility to pathological processes (Doe et al., 2020; Smith et al., 2017), which may reflect the intricate interplay between structural integrity, regional vulnerability, and compensatory mechanisms in response to glaucomatous damage.

This study revealed that among the seven clusters of functional stability differences between the POAG and control groups, a subtle correlation was observed only between the stability of the left visual cortex and VF MD. However, this correlation did not retain significance after multiple comparison correction. Given the absence of significant inter-eye differences in VF MD measurements among POAG patients in this study, and all participants included are right-handed, the subtle difference in correlation may be attributed to the left visual cortex being potentially more sensitive to functional changes that correspond to the severity of visual field deficits. The decreased dynamic stability in the left visual cortex could potentially serve as a predictive factor for disease progression. However, further research involving larger and more diverse cohorts is needed to establish its predictive value with greater certainty and provide a more comprehensive understanding of the lateralization phenomenon and its underlying mechanisms.

Furthermore, we explored the stable abnormal areas of dynamic functional stability identified under different window widths, step sizes, and spatial smoothing kernels, and found that our main results were not affected by the above factors. Currently, sliding window technology is the most commonly used method for exploring functional connections (Hutchison et al., 2013). However, there is still no standardized approach for selecting the window size and step size. The optimal window size (including its size and shape) largely depends on the hemodynamic response of the brain, which is influenced by differences in neurochemical activity and physiological parameters of the cerebrovascular system (Sakoğlu et al., 2010). It has been suggested that the minimum window length should not be less than 1/f_min_, where f_min_ represents the minimum frequency of the time process (Leonardi & Van De Ville, 2015). A window length that is too short may lead to spurious fluctuations, while a window length that is too long may result in the loss of observable dynamic characteristics in the time series. As a consequence, although certain abnormal regions of dynamic connection stability in our study may be challenging to identify due to the factors mentioned above, our main results are somewhat reproducible after controlling for multiple factors.

We have validated the reproducibility of abnormal regions of dynamic functional stability that were identified using different window widths, step sizes, and spatial smoothing kernels. We determined that the core findings of our study remained consistent despite these methodological variations. Presently, sliding window methodology stands as the prevailing approach for exploring functional connections (Hutchison et al., 2013). Nevertheless, a standardized approach for selecting the optimal window size and step size remains elusive. The choice of an appropriate window size (comprising both dimensions and shape) is heavily influenced by the brain's hemodynamic response, which is intricately tied to variations in neurochemical activity and physiological parameters of the cerebrovascular system (Sakoğlu et al., 2010). It has been suggested that the minimum window length should not fall below 1/f_min_, where f_min_ denotes the lowest frequency of the temporal process (Leonardi & Van De Ville, 2015). A window length that is excessively short might yield spurious fluctuations, whereas an overly extended window length could potentially obscure discernible dynamic traits within the time series. Taking into account the aforementioned factors, we devised experimental protocols with various combinations of parameters. Although the detection of specific aberrant regions of dynamic connectivity stability in our study might encounter challenges stemming from various factors, our primary findings exhibit a level of replicability following thorough adjustments for multiple influencing variables.

This study evaluated the dynamic functional changes in primary open-angle glaucoma, analyzed the associations between these changes and visual field alterations, and enhanced accuracy and rationality through data analysis in cortical surface space. The preliminary findings contribute to the study of dynamic functionality in glaucoma patients. However, several limitations should be acknowledged. Firstly, the sample size in our study was relatively small, and therefore, a larger sample from multi-center studies is required to validate the reliability of the results. Secondly, we did not explore the functional stability of subcortical nuclei, leaving room for future investigation. Thirdly, our study is cross-sectional, and further longitudinal studies will be beneficial for investigating the causal factors of alterations in functional stability in POAG patients. Finally, the ophthalmic examination data we collected are not exhaustive, and more indicators need to be obtained from POAG patients to explore their relationship with altered functional stability. Consequently, future comprehensive studies are needed to address these aspects.

## Conclusions

In conclusion, this study has identified a decrease in dynamic functional stability in visual-related brain regions and an increase in dynamic functional stability in attention-related networks. Furthermore, a correlation has been observed between alterations in functional stability within visual areas and visual field defects. This may suggest a potential functional loss in visual brain regions and compensatory mechanisms within attention brain regions. These findings suggest that functional stability may offer a novel perspective to expand current understanding of the neuropathological basis of POAG and could potentially serve as a new approach for early diagnosis and monitoring of POAG progression.

### Supplementary Information

Below is the link to the electronic supplementary material.

Supplementary file1 (DOCX 3912 kb)

Supplementary file2 (XLSX 44 kb)

## Data Availability

The data and materials of this study are available from the corresponding author on reasonable request.
