# Supplementary material for: Altered stability of dynamic brain functional architecture in primary open-angle glaucoma: a surface-based resting-state fMRI study
Source: Brain Imaging Behav. 2023 Oct 19;18(1):44–56. doi: 10.1007/s11682-023-00800-7 (PMC10844345; doi:10.1007/s11682-023-00800-7)
Supplement: Supplementary file 1 — Supplementary file1 (DOCX 3912 kb) [file 11682_2023_800_MOESM1_ESM.docx]

**Supplementary Materials**

**Supplementary Tables**

**Table S1.** Distribution of regions showing significant differences in stability between groups with different sliding-window and spatial smoothing parameters.

**Supplementary Figures**


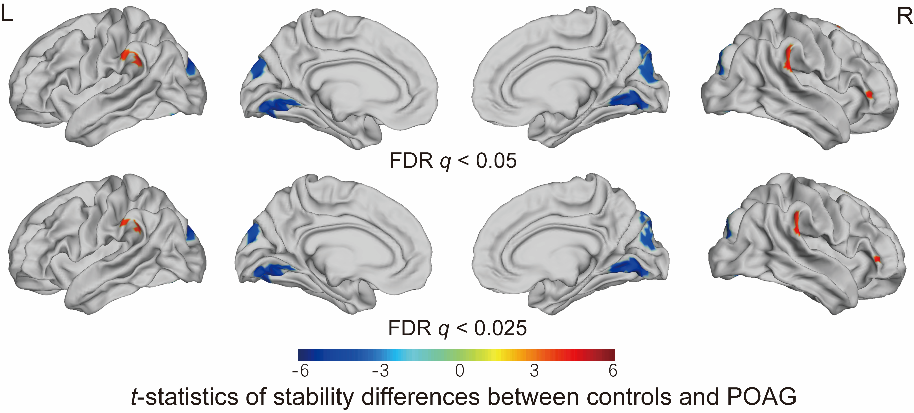


**Figure.S1** Reproducibility of between-group differences of POAG patients and controls under different multiple comparison correction thresholds. Case-control comparison (*t*-map) of dynamic functional stability, with warm colors representing POAG > HC and cold colors representing POAG < HC. L, left; R, right; FDR, false discovery rate; POAG, primary open-angle glaucoma; HC, healthy control.


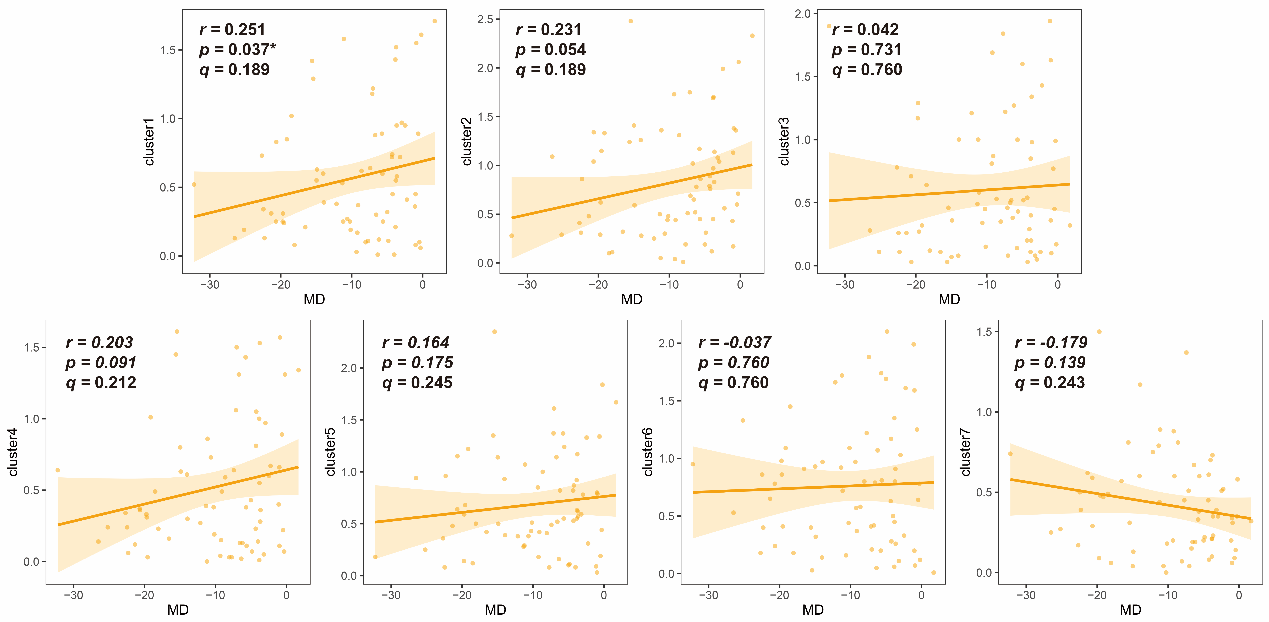


**Figure.S2** Spearman’s correlation analyses between the MD of VF defects and stability value of cluster 1-7 in patients with POAG. In the uncorrected analysis, only cluster 1 values showed a weak positive correlation with MD of VF defects, while other clusters exhibited no significant correlation with MD of VF defects. After FDR correction, all clusters demonstrated no significant correlation with MD of VF defects. MD, mean deviation; VF, visual field; POAG, primary open-angle glaucoma; FDR, false discovery rate.


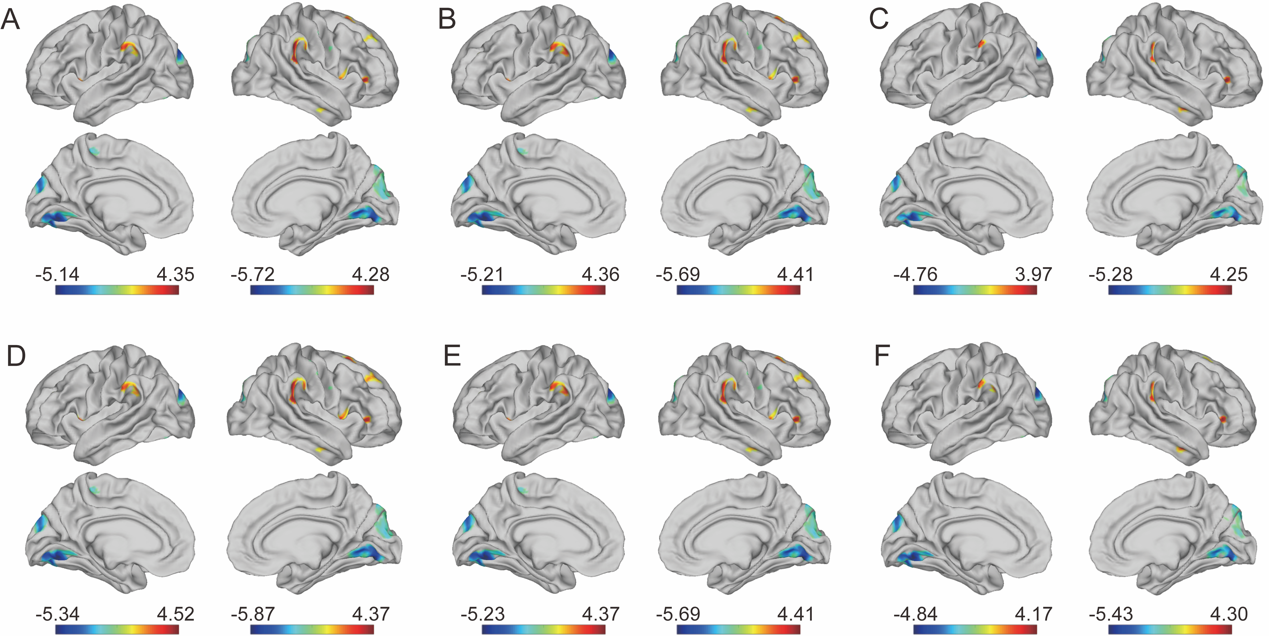


**Figure.S3** The effects of different sliding-window parameters on the functional stability analysis. The sliding-window approach was used to analyze the dynamic functional connectivity with the following parameters: (A) window size = 50 s, sliding step = 2 s; (B) window size = 64 s, sliding step = 2 s; (C) window size = 100 s, sliding step = 2 s; (D) window size = 50 s, sliding step = 4 s; (E) window size = 64 s, sliding step = 4 s; and (F) window size = 100 s, sliding step = 4 s. The primary findings of the between-group comparison can be replicated by performing the analyses of functional stability according to different window size (50 s, 64 s and 100 s) and sliding steps (2 s and 4 s). This indicates that varying sliding-window parameters have no significant influence on the results.


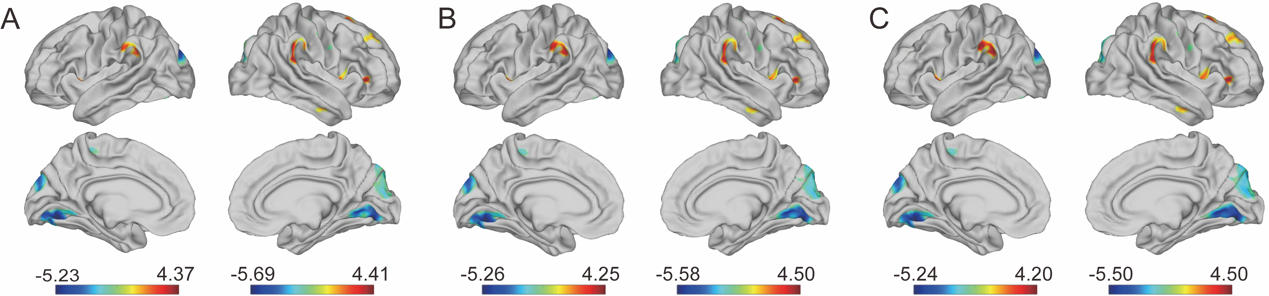


**Figure.S4** The effects of the full­width at half maximum (FWHM) of spatial smoothing on the functional stability analysis. The main findings of the between-group comparison can be replicated by performing the analyses of functional stability according to different FWHM (6 mm, 8 mm and 10 mm), indicating that FWHM does not significantly influence the results.
